# Bacterial Mutations

**Published:** 1914-12

**Authors:** 


**Bacterial Mutations.** G. D. Maynard, "Med. Jour. of South Africa," August, 1914. Some years ago, the editor pointed out that, from purely historic and biologic evidence, the evolution of specific micro-organisms could be given an approximate date. For example, infections occurring spontaneously only or with rare exceptions in human beings, obviously correspond to evolution of germs subsequent to the origin of the human race; infections not indigenous to a given race, as the American Indians, but occurring virulently among them when introduced, must have evolved after the development of man and also after the separation from the parent stock of the given race but long before the rediscovery of that race by the parent stock. Maynard reviews the literature from the more exact standpoint of bacteriologic demonstration. For example, Thelie and Embleton, Lancet, 1913, experimented with the bacillus mycoides, of soil, superficially resembling the anthrax micro-organism but incapable of growth at body temperature and possessing flagella. After sensitizing a guinea pig to this organism by inoculating 20 m. g. dry weight of a culture, intraperitoneal injection caused lesions indistinguishable from anthrax. Cultures recovered from the animal were similarly pathogenic to others. In our article, we alluded to the indirect evidence that the tubercle bacillus was first pathogenic in its bovine form, the human variety being a subsequent evolution, while the bovine type was probably ultimately evolved from the free-growing hay bacillus. The same authors claim to have produced typic, pathogenic tubercle bacilli from both the hay and the semgma bacilli, while Herman Dostal has cultivated the tubercle bacillus into an organism both non-pathogenic and non-acid-fast. (Note: Several years ago, Victor C. Vaughn mentioned a culture at the University of Michigan which had been kept going on artificial media for some years and had lost its virulence.) Rosenow, (sic) "Jour. of Infect. Dis.," January, 1914, (citing other authorities) states that the haemolytic streptococcus, streptococcus viridans, streptococcus of rheumatism, pneumococcus and streptococcus mucosus may be converted into one another, starting at any point and progressing in either direction.

Summarizing, Maynard states that pathogenic development and mutations have been demonstrated in the following groups: colon-typhoid, strepto-pneumococcus, acid-fast or tubercle, mycoides-anthrax, diphtheria. The lessons both as to identification by culture, by agglutinating and analogous reactions, and even in a sanitary sense, are obvious.

